# Sleep Health and Psychological Wellbeing in Adult Women: A Specific Focus on Endometriosis—A Survey Study

**DOI:** 10.3390/jcm14062103

**Published:** 2025-03-19

**Authors:** Elisabetta Baldi, Debora Meneo, Silvia Cerolini, Francesca Gelfo, Chiara Baglioni

**Affiliations:** 1 Department of Human Sciences, Guglielmo Marconi University, 00193 Rome, Italy; e.baldi@unimarconi.it (E.B.); d.meneo@unimarconi.it (D.M.); s.cerolini@unimarconi.it (S.C.); f.gelfo@unimarconi.it (F.G.); 2 IRCCS Fondazione Santa Lucia, 00179 Rome, Italy; 3 Department of Psychiatry and Psychotherapy, Medical Center, Faculty of Medicine, University of Freiburg, 79104 Freiburg, Germany

**Keywords:** endometriosis, sleep health, insomnia, psychological wellbeing, pain

## Abstract

**Background**: Endometriosis is a chronic, oestrogen-sensitive inflammatory disease, which can have a significant impact on women’s wellbeing. Nevertheless, the sleep health of this population has been poorly investigated. This exploratory study aimed at describing sleep health, psychological wellbeing, and prevalence of endometriosis among a sample of female students and at evaluating the impact of endometriosis on sleep health and psychological wellbeing in women suffering from the disease. **Methods**: Women were recruited with a two-stage procedure in a cross-sectional study using online questionnaires: Insomnia Severity Index, Hospital Anxiety and Depression Scale, and Difficulties in Emotion Regulation Scale—Short form. Furthermore, specific questions were used to measure the five dimensions of sleep health: satisfaction, vigilance, efficiency, duration, and timing. **Results**: In the first stage of the enrolment procedure, 1068 students (18–45 years old) responded to the questionnaire, and 8.7% reported a physician diagnosis of endometriosis. Additionally, in the second stage of the enrolment procedure, 75 women were recruited through social media and reported a diagnosis of endometriosis. Then, all women with endometriosis (169) were age-matched with 169 women without the disease. Multivariate regression analyses showed a bidirectional association between sleep health and psychological wellbeing in the matched sample. Independent samples t-test showed that women with endometriosis reported more severe insomnia symptoms and lower psychological wellbeing than controls. Statistically significant differences were also found in global sleep health and satisfaction, vigilance, and efficiency. Among women with endometriosis, pain and anxiety symptoms were bidirectionally associated, while sleep health was significantly associated with disease stage and psychological difficulties. **Conclusions**: Considering and treating sleep difficulties in this population may contribute to an improvement in psychological wellbeing and quality of life.

## 1. Introduction

Women are more susceptible to psychological and sleep difficulties. Insomnia disorder is the most common sleep disorder in the general population [1], and multiple studies and systematic reviews have demonstrated that it is more prevalent in women than men [2,3,4,5]. Several biological and psychosocial factors seem to explain the higher prevalence of insomnia and poor sleep health in women. Biological factors include hormonal differences, changes in hormone levels over a woman’s life, and comorbid medical conditions [5,6]. Psychosocial factors encompass anxiety, depression, and stress related to gender role expectations [6,7,8]. Although much of the higher prevalence of insomnia and other mental health issues in women may be explained by the hormonal or psychosocial changes associated with major life transitions (e.g., puberty, pregnancy, and menopause) [9], some of the gender differences may partially result from the higher prevalence of pain [10] and the presence of chronic gynaecological conditions in women.

Endometriosis is a chronic gynaecological disease defined by the presence of endometrial-like tissue (lesions) outside the uterus [11]. The displacement of endometrial tissue into ectopic regions (e.g., ovaries, peritoneum, rectum, urinary bladder, uterosacral ligaments, abdominal wall, diaphragm, etc.) causes a wide range of symptoms, including chronic pains and infertility. Endometriosis is associated with higher risk of infertility [12], with studies suggesting that up to 50% of infertile women have endometriosis and that 30% to 50% of women with endometriosis are infertile [13,14,15]. Globally, approximately 10–15% of women of reproductive age experience endometriosis [16]. However, although it is a widespread disease, it is often misdiagnosed, leading to delayed medical recognition [17,18,19] and tardive treatment.

The disease can significantly impact psychological health, leading to increased stress, fatigue, limited daily life activities, impaired work productivity, and mental health disorders [20,21]. Many studies have also demonstrated the influence of this disease on mental health, showing a significant association with depressive and anxiety-related disturbances [22]. Moreover, women with endometriosis have a significantly higher risk of mental disorders than healthy ones [23,24,25], and endometriosis-related pain is considered a significant contributor to poorer psychological wellbeing [26,27,28].

While pain and infertility have received more attention, sleep disturbances in women with endometriosis have been less investigated [29], despite the relevance of sleep for psychological wellbeing. Recently, a systematic review by Sumbodo et al. [29] on the relationship between sleep disturbances and endometriosis found that sleep disturbances are significantly more common in women with endometriosis compared to healthy women. Almost all included studies reported a significant positive association between endometriosis and sleep disturbances, specifically poor sleep quality, daytime sleepiness, and insomnia [30,31,32,33,34,35,36]. The high prevalence of sleep difficulties and insomnia in women with endometriosis is often attributed to hormonal fluctuations, mood disorders, or gynaecological dysfunctions [37].

The rationale for the association between endometriosis and sleep disturbances may be multifaceted. On the one hand, chronic pain, which is one of the core symptoms of the disease, directly exacerbates sleep difficulties [38]. On the other hand, sleep impairments increase inflammation and the experience of pain [39]. That is, there is a bidirectional association between pain and sleep disturbances, which can result in the creation of a vicious cycle, with pain causing sleep disturbance that increases inflammation, leading to more severe pain [37]. Furthermore, an experimental study showed that the association between sleep and inflammatory markers is stronger in women than in men [40], suggesting that women with both insomnia and endometriosis complaints are more susceptible to symptoms of pain [37].

Moreover, fluctuations in reproductive hormones can affect sleep patterns [41], since they regulate many homeostatic functions, including the sleep–wake cycle [9]. The elevated secretion of various hormones (oestradiol and progesterone) during specific women’s life stages can significantly influence both the circadian and the homeostatic processes of sleep regulation, resulting, for example, in delayed sleep timing during puberty [42], alterations in sleep architecture and sleep difficulties during pregnancy [41,43], and sleep fragmentation during menopause [44]. Since endometriosis is a chronic oestrogen-dependent pathology, hormonal changes in women suffering from the disease can intensify, thus contributing to sleep disruption [41,45]. Beyond the physiological mechanisms, psychological processes may contribute to the worsening of sleep. On the one hand, sleep disturbances may result in irritability, decline in motivation, and difficulties effectively managing negative emotions [46]. Conversely, emotional dysregulation and other changes in emotional processes can raise psychophysiological arousal, which can interfere with sleep [47,48,49], raising the possibility of a vicious cycle between sleep disturbance and emotional dysregulation [50]. In sum, the presence of sleep difficulties in endometriosis could be a risk factor both for the worsening of disease symptoms and for the onset of psychopathology.

Good sleep is essential for good health [51], while nonoptimal sleep health can impair the quality of life and daytime functioning, compromising physical and mental health. Sleep health has been defined as a positive dimensional concept, that is, a continuum ranging from optimal sleep health to poor/pathological sleep [51]. It is characterised by five dimensions: (a) satisfaction with sleep, (b) vigilance (or daytime alertness or sleepiness), (c) sleep timing and regularity, (d) sleep efficiency, and (e) sleep duration. Alterations in any of these dimensions are associated with poor physical and mental health outcomes, in both clinical and healthy populations [51]. Early identification of these alterations can allow a preventive approach to the onset of chronic sleep behavioural disorders and their psychopathological consequences [52,53,54]. A greater understanding of the role of sleep health in women affected by endometriosis can pave the way for prevention and treatment interventions aimed at improving quality of life and psychophysical wellbeing.

Considering this framework, we conducted an exploratory study to investigate sleep health and psychological wellbeing in adult women, along with the prevalence of endometriosis, and to evaluate the impact of endometriosis on mental and sleep health, comparing a clinical sample of women with an age-matched healthy sample.

### Aims of the Present Study

This exploratory study had three principal aims:

1. To explore and describe sleep health, psychological wellbeing, and prevalence of endometriosis among a sample of adult women;
2. To evaluate the impact of endometriosis on sleep health and psychological wellbeing in adult women, examining significant differences between women affected by endometriosis and women of the same age without a diagnosis;
3. To examine the relationship between sleep health, psychological wellbeing, and clinical characteristics of the disease among women with endometriosis.

This study aims first to offer a snapshot of the relationship between sleep health and psychological wellbeing and to examine the prevalence of endometriosis among a large sample of adult women and then focus specifically on the relevance of the topic in women with endometriosis. The main hypothesis is that women with endometriosis would have significantly worse sleep health and psychological wellbeing than women without the diagnosis. Furthermore, we hypothesised that, in the endometriosis group, disease-related symptoms and women’s wellbeing would impact each other. Overall, the present paper aims to provide a greater understanding of how sleep health is associated with psychosocial factors and symptoms related to the disease.

## 2. Materials and Methods

This study was carried out in compliance with the Declaration of Helsinki and, on 19 June 2023, the Guglielmo Marconi University Ethics Committee for Psychological Studies approved the study protocol (approval code: 20230619). All participants provided written informed consent, and confidentiality in data collection was preserved.

### 2.1. Enrolment Procedure

A two-stage enrolment procedure was implemented to pursue the three aims of the present study. Firstly, to investigate sleep health, psychological wellbeing, and prevalence of endometriosis in adult women, an e-mail was sent by the administration to all enrolled students at Guglielmo Marconi University. Both women who reported a physician diagnosis of endometriosis and women without the diagnosis responded to the questionnaire and were enrolled. Since the university administration had no way of knowing who had completed the questionnaire, participation in the study was entirely voluntary. Then, to pursue the second and the third aims, it was necessary to expand the sample of women with endometriosis to enhance statistical power for the comparison (for a priori power analysis for sample size, see Section 2.3). Consequently, the second stage of the recruitment strategy included posting advertisements on social media groups of women with endometriosis. Participating women were also invited to share the invitation to the study with their acquaintances. No compensation was provided to participants, either in terms of money or university credits.

Inclusion criteria for all women were (a) age between 18 and 45 years and (b) signed informed consent. Women were excluded if they reported physiological menopause. Having a gynaecological disease, apart from endometriosis, was an exclusion criterion. Women with an induced or surgical menopause due to endometriosis were not excluded, as this is a therapeutic strategy to control endometrial growth.

Recruitment was continuous from June to December 2023, and both groups completed the same questionnaires (about 15 min long), except for a clinical information module related to endometriosis, which could only be filled in by women with the diagnosis. Indeed, women were asked if they had ever received a diagnosis of endometriosis by a physician and those who answered affirmatively could continue by filling out the clinical information module. Before completing the questionnaires, interested women were asked to read the Information for Participation and provide their consent to participate through Informed Consent. Only women expressing their consent to participation could continue with the questionnaire.

### 2.2. Survey Structure

Participants enrolled in the study completed a set of questionnaires developed on the Google Forms^®^ platform. The online form was composed of the following sections and questionnaires:

Demographic information: age, relationship status, employment status, and educational level.

Clinical information (only for women with endometriosis): clinical information included age at the time of first diagnosis of endometriosis, time from the onset of symptoms, any previous surgical interventions, and current stage of the disease. The stage, and thus the severity level of the disease, was categorised according to the American Society for Reproductive Medicine (ASRM) classification system, which is currently the best-known classification of endometriosis and is one of the most widely used throughout the world [55]. It classifies the severity of endometriosis into four grades, using points that correspond to the size of the endometriosis lesions in the peritoneum and ovaries: minimal (stage I), mild (stage II), moderate (stage III), and severe (stage IV) stages. Moreover, women were asked if they were taking any prescription medications (hormonal therapies) or other medications (analgesics). In addition, three visuo-analogue scales were used to rate from 0 = “none” to 10 = “very severe” dysmenorrhea, chronic pelvic pain, and dyspareunia, which are three common symptoms of the disease.

Insomnia Severity Index (ISI) [56,57]: a 5-point Likert scale composed of 7 items, with a total score ranging from 0 to 28. It gives a total score of the severity of insomnia symptoms and can be interpreted as follows: not clinically relevant insomnia symptoms (0–7); subthreshold insomnia (8–14); moderate insomnia (15–21); and severe insomnia (22–28). Each question of the questionnaire specifically referred to the previous week and the previous month.

Reduced Morningness–Eveningness Questionnaire (rMEQ) [58]: a 5-items scale evaluating circadian preference. The sum of the items leads to a total score from 4 to 25. The scores are interpreted as evening typology (4–10), intermediate typology (12–17), and morning typology (19–25).

Hospital Anxiety and Depression Scale (HADS) [59,60]: a 14-item questionnaire composed of two subscales: anxiety symptoms (7 items) and depressive symptoms (7 items). The values for each subscale are the sum of the responses to the respective items (0–3). The scores for each scale are interpreted as: normal score (0–7), mild (8–10), moderate (11–15), and severe (16–21). Each question in the questionnaire was referred to the previous month and the previous week specifically.

Difficulties in Emotion Regulation Scale—Short form (DERS-SF) [61,62]: the short form of DERS [63] for the multidimensional assessment of emotion regulation was used. The DERS-SF is composed of 18 items addressing the evaluation of the frequency of behaviours, beliefs, or experiences related to emotions, with scores ranging from 1 = “almost never” to 5 = “almost always”. The scale investigates 6 dimensions of emotion regulation: acceptance of emotional responses; engagement in goal-directed behaviours; control of impulsive behaviours; emotional awareness; access to emotional regulation strategies; and emotional clarity. Higher total scores indicate greater difficulties in emotion regulation.

A specific set of 21 questions was used to explore the five dimensions of *sleep health* identified by Buysse [51]: satisfaction (5 items, e.g., “Do you spend a lot of time feeling completely awake in the morning?”), vigilance (4 items, e.g., “During the day, do you feel that you don’t have enough energy or motivation to complete tasks?”), timing (7 items, e.g., “What is your usual bedtime before a work or school day?”), efficiency (3 items, e.g., “How long does it take for you to fall asleep?”), and duration (2 items, e.g., “How many hours do you sleep on average the nights before a work or school day?”). The scores were calculated by summing the items of each dimension. The scoring of the efficiency and duration scales is carried out according to international guidelines [64]. The sum of the 21 items gives a total sleep health score, with higher scores indicating better health.

### 2.3. Data Analysis

The G-Power v. 3.1 software [65] was used to calculate the a priori sample needed for the study. The power calculation was performed for the part of the study evaluating the impact of endometriosis on sleep health and psychological wellbeing. We considered sleep health as the primary outcome. The effect size could not be estimated because no previous study had employed a comprehensive measure of sleep health like the one used in this study. It was thus set as a medium value (d = 0.50). To obtain sufficient power (0.80) and α = 0.05, a minimum sample of 128 participants (64 women with endometriosis and 64 healthy women) was estimated to be adequate for independent samples t-tests, 168 for linear correlations, and 236 for multivariate linear regression models. The minimum sample size for adequate power of all analyses was thus set at 240.

Endometriosis prevalence, along with participants’ demographics and clinical information, were reported in detail, together with information on the examined indicators. The percentage for categorical variables and the mean value with standard deviation for continuous variables were reported through descriptive analyses. Significant differences in the variables between women with and without a diagnosis of endometriosis were analysed through t-tests with Bonferroni correction and chi-square tests. The associations of the examined psychological variables were analysed through Pearson’s correlations and linear regression. Four multivariable linear regression models were performed to detect the variables associated with (a) insomnia; (b) sleep health; (c) anxiety symptoms; and (d) depressive symptoms. In this way, it was possible to test the impact of insomnia and sleep health on psychological wellbeing and, vice versa, the impact of psychological wellbeing on insomnia and sleep health. Statistical models were first performed on the entire sample, including the diagnosis of endometriosis as an independent variable. All models included age and chronotype as controlling variables, given their association with sleep [66,67]. Predictors in the models were included if they showed significant correlations with the outcome of each model. Regression analyses were then conducted for endometriosis participants, including disease-specific variables (pain level and disease stage) as predictors. A final model was performed with pain as an outcome, averaging the score of the three pain-related items: pelvic pain, dysmenorrhea, and dyspareunia. For all analyses, the level of statistical significance was defined by *p* < 0.05. The R software (version 4.3.3, The R Foundation for Statistical Computing 2021, https://www.r-project.org/ accessed on 14 March 2025) was used for all statistical analyses.

## 3. Results

### 3.1. Demographic and Clinical Characteristics of the Sample and Endometriosis Prevalence

A total of 1171 women responded to the online questionnaire. Three women reported physiological menopause and were excluded from the analyses, resulting in a total sample of 1168 women (18–45 years old, 31.15 ± 7.63). The majority of the sample (*n* = 1078, 30.72 ± 7.57 years old) consisted of female students who were enrolled in Guglielmo Marconi University, an online university. The majority of them were married or cohabiting, employed, and had completed high school. Among students, the prevalence of self-reported diagnosis of endometriosis was 8.7%, approximating the prevalence found in the general female population (10%) [16]. Among the sample of students, average scores fell into the subthreshold range for insomnia (9.67 ± 5.94) and borderline range for anxiety (8.56 ± 4.27) symptomatology, while depressive symptoms were non-clinically relevant. They also presented an average score of 40.71 (±13.57), out of a maximum of 90, in difficulties in emotion regulation. Furthermore, the sample received an average global sleep health score of 31.23 (±7.31) out of a maximum score of 55, which indicates better sleep health. Women reported lower scores on two dimensions compared to the maximum score: timing and satisfaction. The demographic and clinical characteristics of the sample of students are presented in Supplemental Table S1.

Among all responders (*n =* 1168), 169 women (14.5%) reported a diagnosis of endometriosis (19–45 years old, 34.76 ± 7.04) and 999 were healthy women, without a diagnosis of endometriosis or any other gynaecological disease. The 169 women who reported the diagnosis of endometriosis were age-matched using the Propensity Score Matching (PCM) method with 169 women among the 999 without the disease. The demographic and clinical characteristics of the matched sample (*n* = 338) are shown in Table 1. The two groups did not present any statistical difference in employment status (*p* = 0.641), relationship status (*p* = 0.122), or educational level (*p* = 0.309). In terms of the clinical features associated with endometriosis, the average age at the moment of diagnosis was 27.85 ± 6.85 years. Most women with endometriosis reported they first showed symptoms of the disease 10 years before the study, more than half underwent surgery, and one third reported a severe level of the disease (IV). Most of the women affected by endometriosis (65.7%) took hormonal therapy, which was, on average, associated with a worsening of psychological health.

### 3.2. Impact of Endometriosis on Sleep and Psychological Wellbeing

Sleep and psychological data of the two groups are shown in Table 2. Women with endometriosis reported a higher prevalence of severe insomnia (7.1%), anxiety (17.8%), and depressive (7.7%) symptoms compared to the control group. A chi-square test of independence was conducted to test whether the distribution of moderate to severe insomnia, anxiety, and depressive symptoms differed between the two groups. The tests were significant, indicating a higher prevalence of clinically relevant symptoms of insomnia (χ^2^ = 14.993, *p* < 0.001), anxiety (χ^2^ = 12.084, *p* < 0.001), and depression (χ^2^ = 16.626, *p* < 0.0001) in the endometriosis group compared to the control group. The distribution of chronotypes was similar between groups (*p* = 0.131) and approximated the normal distribution found in the general population [68,69]. Independent samples t-test showed that women with endometriosis reported significantly higher insomnia symptoms (12.96 ± 6.26) than healthy controls (9.47 ± 5.99; *p* < 0.001). Statistically significant differences were also found in global sleep health, with women affected by endometriosis showing poorer sleep health (27.66 ± 7.42) than controls (31.96 ± 7.28; *p* < 0.001). Also, women with endometriosis reported significantly lower scores in specific dimensions of sleep health (satisfaction, vigilance, and efficiency; *p* < 0.001) compared to controls, except for timing and duration. Using the “National Sleep Foundation” guidelines to divide sleep duration into ranges [64], no significant differences between groups were found on weekdays or weekends. Moreover, women with endometriosis reported significantly higher levels of anxiety (10.53 vs. 7.83) and depressive (8.75 vs. 6.28) symptoms and more difficulties in emotion regulation (45.02 vs. 38.20; *p* < 0.001) compared to the control group.

### 3.3. Comparisons Between Women with Endometriosis with Clinical and Nonclinical Insomnia Symptoms on Sleep Health and Psychological Wellbeing

Based on the ISI cut-off of 14 [56,70], women with endometriosis were divided into two groups: those reporting clinically relevant symptoms of insomnia (moderate to severe) and those reporting subthreshold or not clinically significant symptoms. The two groups’ differences on clinical, psychological, and sleep health indicators are displayed in Table 3. According to independent samples t-tests, women with clinical symptoms of insomnia exhibited considerably greater levels of anxiety (*p* < 0.001) and depression (*p* = 0.001), as well as greater difficulty regulating their emotions (*p* < 0.001). They also reported lower global sleep health (*p* < 0.001) and poorer sleep health in single dimensions (satisfaction, vigilance, efficiency, and duration). Women with endometriosis with clinical insomnia symptoms reported more pain, especially pelvic pain (*p* < 0.05) and dyspareunia (*p* < 0.001), compared to women without clinical insomnia symptoms.

### 3.4. Relationship Between Sleep Health and Psychological Wellbeing

Pearson correlations between questionnaire scores were calculated among the 338 women (Table 4). ISI scores were negatively correlated with total sleep health scores (r = −0.67, *p* < 0.001). Moderate to strong positive and significant correlations were found between insomnia symptoms and anxiety (r = 0.51, *p* < 0.001), depressive symptoms (r = 0.45, *p* < 0.001), and emotion regulation difficulties (r = 0.47, *p* < 0.001). Better sleep health was similarly negatively associated with anxiety (r = −52, *p* < 0.001) and depressive (r = −0.49, *p* < 0.001) symptoms and emotion regulation (r = −0.53, *p* < 0.001).

### 3.5. Bidirectional Associations Between Sleep Health and Psychological Wellbeing

Four multivariable linear regression models (Table 5) were performed on the matched sample (*n* = 338) to detect the variables associated with (a) insomnia; (b) sleep health; (c) anxiety symptoms; and (d) depressive symptoms. Anxiety symptoms (*p* < 0.001), emotion regulation (*p* < 0.001), and belonging to the endometriosis group (*p* = 0.039) significantly predicted insomnia symptoms (R^2^ = 0.34, F _(6, 331)_ = 27.85, *p* < 0.001); also, belonging to the endometriosis group (*p* = 0.044), anxiety (*p* = 0.001), depressive (*p* = 0.014) symptoms, and emotion regulation (*p* < 0.001) were significantly associated with global sleep health (R^2^ = 0.45, F _(6, 331)_ = 45.27, *p* < 0.001).

To reduce multicollinearity in models predicting anxiety and depression, ISI and sleep health scores were entered individually in two different models. This allowed the unique impact of insomnia symptoms and sleep health on psychological wellbeing to be assessed. Both insomnia symptoms and sleep health contributed to anxiety symptoms, together with depressive symptoms and emotion regulation difficulties (model with insomnia: R^2^ = 0.59, F _(6, 331)_ = 79.25, *p* < 0.001; model with sleep health: R^2^ = 0.58, F _(6, 331)_ = 76.79, *p* < 0.001). Insomnia symptoms did not contribute to depressive symptoms, while sleep health significantly did; in both models, depressive symptoms were associated with anxiety and emotion regulation difficulties (model with insomnia: R^2^ = 0.55, F _(6, 331)_ = 66.87, *p* < 0.001; model with sleep health: R^2^ = 0.56, F _(6, 331)_ = 68.77, *p* < 0.001).

### 3.6. Association Between Disease-Specific Factors and Wellbeing

Among women with endometriosis, univariate regression models were performed to estimate the direct effect of predictors on each outcome. Univariate regression models are reported in Supplemental Table S2. Emotion regulation, anxiety, depression, and average pain were significant predictors of insomnia symptoms in univariate models; together with disease stage IV, they also predicted global sleep health. In multivariable regression models (Table 6 and Table 7), only anxiety symptoms remained a significant predictor of insomnia (β = 0.41, *p* = 0.006), while anxiety symptoms (β = −0.45, *p* = 0.007), emotion regulation (β = −0.12, *p* = 0.006), and disease stage IV (β = −3.41, *p* = 0.020) significantly predicted sleep health (R^2^ = 0.51, F _(8, 118)_ = 15.04, *p* < 0.001).

In models predicting anxiety, depression, and pain, ISI and sleep health scores were entered individually into two different models to assess the unique impact of insomnia symptoms and sleep health on psychological wellbeing and endometriosis-related pain. Depressive symptoms, emotion regulation, sleep health, insomnia symptoms, and pain average significantly predicted anxiety symptoms in univariate models. These variables, except for sleep health, remained significant predictors in multivariable regression models (model with insomnia: R^2^ = 0.52, F _(4, 159)_ = 43.26, *p* < 0.001; model with sleep health: R^2^ = 0.51, F _(4, 159)_ = 41.55, *p* < 0.001).

Anxiety symptoms, emotion regulation, sleep health, insomnia symptoms, average pain, and disease stage IV were significantly associated with depressive symptoms in univariate models. In multivariable regression models, only anxiety symptoms and emotion regulation remained significant predictors of depressive symptoms (model with insomnia: R^2^ = 0.46, F _(7, 119)_ = 14.71, *p* < 0.001; model with sleep health: R^2^ = 0.47, F _(7, 119)_ = 15.21, *p* < 0.001).

Considering disease-related pain, sleep health, anxiety, depression, insomnia symptoms, disease stages (II, III, and IV), emotion regulation, and having symptoms for 10 years were significant contributors in univariate models. In multivariable regression models, only anxiety and disease stages remained significant predictors of pain (model with insomnia: R^2^ = 0.27, F _(10, 116)_ = 4.28, *p* < 0.001; model with sleep health: R^2^ = 0.27, F _(10, 116)_ = 4.36, *p* < 0.001).

## 4. Discussion

Endometriosis is a chronic gynaecological disease associated with an impairment of psychological wellbeing, impaired health-related quality of life, and mental health disorders [20,21]. The present study aimed at assessing sleep health and psychological wellbeing in women and the impact of endometriosis. We found a prevalence of endometriosis of 8.7% in a sample of adult women, confirming the disease prevalence found in previous studies [16]. Descriptive analyses were used considering all students responding to the questionnaire (*n =* 1078) to explore the distribution of sleep health dimensions and psychological outcomes. Considering this sample, women reported elevated, but nonclinical, levels of anxiety and insomnia but not of depression. This might be a result of the sample being exclusively composed of women. The prevalence of anxiety and insomnia disorders in this group may have been higher than in the general population because it is generally known that these conditions are more common in women than in men [1,71]. Furthermore, as this was a survey study, women who experienced these issues would have been more likely to respond to the questionnaire. Emotion regulation scores were in line with the results of previous studies on university students [72]. Descriptive analyses were used to have a first snapshot of the distribution of the five dimensions of sleep health [51] in adult women. Though this was an exploratory aim of the present work, still some observations could be reported. In the sample of students, the average global sleep health scores were quite far from optimal sleep health. Women reported lower scores in satisfaction and timing compared to the maximum scores, but it is not possible to compare the scores with a reference due to the lack of a cut-off and information about how these dimensions are distributed in the general population. Gender differences in sleep health dimensions should be deepened and better understood by future research.

Then, women with endometriosis were compared to healthy women. A higher prevalence of moderate to severe symptoms of anxiety and depression, as well as more difficulties in emotion regulation, were found in women with endometriosis compared to the age-matched control group and confirmed our primary hypothesis of higher insomnia symptoms and lower psychological wellbeing in this clinical sample. This is consistent with previous studies showing that women with endometriosis experience poorer sleep quality, more daytime sleepiness, and higher insomnia severity compared to healthy women [29,73]. To our knowledge, no study has yet examined the association between sleep health dimensions in women with endometriosis. Early identification of alterations in these dimensions could be important to prevent the onset of chronic sleep behavioural disorders, such as insomnia disorder, and their psychopathological consequences [52,53,54]. By examining the different dimensions of sleep health, we found that women with endometriosis, compared to controls, displayed significantly lower scores in satisfaction, vigilance, and efficiency, which are the crucial dimensions in insomnia disorder. Interestingly, no statistical differences were found in timing and duration. People with insomnia disorder experience poor sleep efficiency, with difficulties in falling and/or staying asleep, dissatisfaction with their sleep, and a great daytime impairment. This finding may highlight that good sleep is not only given by an adequate duration and regular sleep timing, endorsing the dimensional construct of sleep health. The subjective perception of one’s sleep is important. It is essential, for example, how someone wakes up in the morning (satisfaction with the sleep from the previous night), whether or not they fell asleep quickly and slept through the night (efficiency), and how they feel the next day (vigilance).

Comparing women with endometriosis with and without clinical insomnia symptoms, we found higher anxiety and depressive symptoms and more difficulties in emotion regulation in women with clinical insomnia symptoms. They also experienced poorer global sleep health and reported lower scores in individual sleep health dimensions, i.e., satisfaction, vigilance, efficiency, and duration. Women with clinical insomnia symptoms also reported more pain, confirming previous studies [37]. These results suggest a strong association between insomnia and mental health, with insomnia potentially leading to poorer psychological health and being a risk factor for psychopathology [52,53,54]. This finding has also been confirmed by our correlation analysis, which showed strong positive correlations between insomnia symptoms and anxiety, depressive symptoms, and emotion regulation difficulties.

In multiple regressions on the matched sample, anxiety and emotion regulation significantly predicted both insomnia symptoms and sleep health in the expected direction. Moreover, as for our hypothesis, we found a bidirectional association between psychological health and sleep. The scientific literature provides much evidence of this bidirectional relationship, with mental health influencing sleep patterns and sleep disturbances exacerbating psychiatric symptoms. It is well known that sleep difficulties and insomnia may increase the risk of developing various mental health conditions [52,53,54] and, in turn, many mental disorders have many consequences on sleep patterns [74].

On the one hand, sleep disorders can exacerbate existing psychiatric conditions, increase the risk of developing new mental disorders, and contribute to the persistence and severity of symptoms [74,75]. On the other hand, mental disorders may trigger sleep difficulties and disturb sleep patterns. Furthermore, several epidemiological studies have shown that mental health problems and sleep difficulties frequently co-occur [75]. Several studies have suggested the bidirectional relationship between sleep and mental disorders, especially depression [76,77,78] and anxiety [76,79].

In women with endometriosis, insomnia symptoms, and sleep health were also associated with the average level of pain and the stage of the disease. However, anxiety symptoms result as a stronger predictor of both insomnia and sleep health. This result is consistent with previous research, which has found a bidirectional association between sleep disturbances and anxiety [76,79] and a great prevalence of anxiety in women with endometriosis. A recent systematic review has shown a prevalence of anxiety symptoms ranging from 11.5 to 87.5% in this clinical population and a significant impairment of their quality of life [80]. Thus, anxiety is an important issue to consider in this population.

The psychological health and level of disease-related pain of women with endometriosis was, on the other hand, strongly impacted by insomnia and sleep health. Chronic pain is the core symptom of endometriosis, and it plays a central role in this pathology. A recent cross-sectional study found that, despite treatment, about 50% of women experience pain, and pain is associated with negative effects on several areas of daily life, including standing, walking, sitting, sports activities, family and domestic responsibilities, sexuality, social functioning, and professional life [81]. Targeting sleep difficulties and insomnia may potentially be beneficial for pain, which is essential for improving the quality of life in this population. Overall, these findings confirmed our secondary hypothesis of a reciprocal association between sleep health and psychological wellbeing.

This exploratory study provided further evidence of the association of sleep with both physical and psychological wellbeing in a chronic pathology. The link between sleep and mental health has also been demonstrated by several studies, systematic reviews, and meta-analysis, which have supported that targeting sleep difficulties can also improve mental health [82,83,84]. Nevertheless, the role of sleep and the presence of sleep behavioural disorders in women affected by endometriosis have received little attention in basic research and clinical practice, which has led to the underdiagnosis and undertreatment of sleep disorders. The most prevalent sleep behavioural disorder is insomnia disorder in both general and clinical populations [1], and it is more common in women with endometriosis compared to healthy ones. Considering that Cognitive Behavioural Therapy for Insomnia (CBT-I) disorder is the first-line treatment for insomnia [85,86], this treatment could be potentially beneficial for women affected by this pathology, leading to an improvement in their sleep complaints and mental health as well. The assessment of sleep in general, and insomnia in particular, should be an integral part of a comprehensive assessment and screening strategy by mental health and primary care providers [82]. Furthermore, given that insomnia can be a risk factor in the development of mental disorders [52,53,54], treatment of insomnia should be considered both as treatment and as preventive mental health care. Considering that sleep disorders can affect and exacerbate physical (e.g., chronic pain) and mental health, treatment of sleep difficulties and insomnia disorder should be added to the clinical management of endometriosis, with potential benefits on health-related quality of life, psychological distress, and chronic pain in this clinical population. Future research should further investigate the role of sleep and sleep disturbances in endometriosis and should test the effectiveness of CBT-I in improving insomnia- and endometriosis-related symptomatology in women with this comorbid condition.

### Limitations

To the best of our knowledge, this is the first study providing insight into sleep health in endometriosis and its association with psychological health and clinical characteristics. However, this study is not free of limitations. Firstly, the study is a survey study, and the data are based on self-report, which is known to be limited by recall biases. Furthermore, the diagnosis of endometriosis was directly asked to participants (i.e., women were asked if they had received a diagnosis by a physician) and not derived by a physician or from medical records. Moreover, we did not collect information about which type of hormonal therapy the participants were taking and which type of surgery they underwent. In the future, the involvement of a physician in the study could be useful to collect more certain, accurate, and specific information about the diagnosis, clinical characteristics, and treatment of the disease. Additionally, we did not address the diagnostic delay of endometriosis, which is a major concern in its treatment. We focused only on time the women spent experiencing symptoms of the disease, which is a crucial factor to consider when studying psychological health. Further studies should focus more on diagnostic delay to point attention to this societal and public health issue. Moreover, we referred to the ASRM system to classify the severity level of the disease. However, other classification systems, such as the ENZIAN system [87], may give further information about the disease. Future research should address this aspect, also considering other classification systems of endometriosis. Furthermore, the cross-sectional design hinders causal interpretations. The sample size for comparisons was similar to previous studies [30,32] and higher than estimated to be necessary in a priori power analysis. Nonetheless, caution should be paid when generalising results, as the sample came mainly from university students. Future research on more diverse and larger samples is recommended. Lastly, we used a set of questions to investigate sleep health in a more comprehensive way, which was not validated. In the future, the investigation of sleep health in women with endometriosis could use a combination of self-report, sleep diary, and physiological assessment (e.g., actigraphy and polysomnography). Moreover, prospective designs, including ecological-momentary assessment (EMA) designs, can further increase our knowledge of the reciprocal association between sleep health, psychological health, and pain in this population.

## 5. Conclusions

Studying the relationship between sleep and mental health in women is necessary and of utmost importance [41]. Endometriosis has a major impact on many areas of life for women who are affected by it. Despite the relevance of sleep for both physical and mental health, sleep disturbances in women with endometriosis have been poorly investigated. Women with endometriosis have a greater impairment in psychological wellbeing and sleep health, especially in those dimensions that are crucial in insomnia disorder. Furthermore, psychological health, disease-related pain, and sleep health are reciprocally associated. A greater understanding of the role of sleep health in women with endometriosis could allow prevention and treatment interventions to improve quality of life and psychophysical wellbeing. In conclusion, targeting sleep difficulties with psychological intervention is a promising way to improve and enhance women’s wellbeing and mental health disease-related aspects.

## Figures and Tables

**Table 1:** Demographic and clinical characteristics of the matched sample (= 338).

	Endometriosis (*n* = 169)	Controls (*n* = 169)	Total (*n* = 338)	*p* Value
**Age** Mean ± SD Age range	34.76 ± 7.04 19.00–45.00	34.76 ± 7.04 19.00–45.00	34.76 ± 7.02 19–45	1.000 ^a^
**Employment** Employed Not employed Student Student-worker Other	126 (74.6%) 17 (10.1%) 23 (13.6%) 3 (1.8%) 0 (0.0%)	122 (72.2%) 13 (7.7%) 30 (17.8%) 3 (1.8%) 1 (0.6%)	248 (73.4%) 30 (8.9%) 53 (15.7%) 6 (1.8%) 1 (0.3%)	0.641 ^b^
**Relationship status** Married or cohabiting In a relationship Divorced or separated Single	91 (53.8%) 28 (16.6%) 8 (4.7%) 42 (24.9%)	81 (47.9%) 46 (27.2%) 8 (4.7%) 34 (20.1%)	172 (50.9%) 74 (21.9%) 16 (4.7%) 76 (22.5%)	0.122 ^b^
**Education** Middle school diploma High school Bachelor’s degree Master’s degree/single-cycle degree Post-graduate master’s degree PhD Other	3 (1.8%) 75 (44.4%) 39 (23.1%) 31 (18.3%) 16 (9.5%) 3 (1.8%) 2 (1.2%)	0 (0.0%) 78 (46.2%) 47 (27.8%) 30 (17.8%) 13 (7.7%) 1 (0.6%) 0 (0.0%)	3 (0.9%) 153 (45.3%) 86 (25.4%) 61 (18.0%) 29 (8.6%) 4 (1.2%) 2 (0.6%)	0.309 ^b^
**Age at the moment of diagnosis** Mean ± SD	27.85 ± 6.85			
**Time from symptoms onset** More than 10 years ago 4–10 years ago 0–3 years ago	101 (59.8%) 47 (27.8%) 21 (12.4%)			
**Surgery (any type)** Yes No	92 (54.4%) 77 (45.6%)			
**Severity level of the disease** Minimal (I) Mild (II) Moderate (III) Severe (IV) Not sure	23 (13.6%) 24 (14.2%) 35 (20.7%) 49 (29.0%) 38 (22.5%)			
**Diagnosed with medical and/or psychological comorbidities**	65 (38.5%)			
**Diagnosed sleep disturbances** Chronic insomnia Sleep apnoea Sleep–wake cycle circadian disturbance Hypersomnolence Women with sleep disturbances in comorbidity	10 (5.9%) 4 (2.4%) 6 (3.6%) 4 (2.4%) 5 (2.6%)			
**Hormonal therapy (any type)** Less than 6 months More than 6 months None	15 (8.9%) 96 (56.8%) 58 (34.3%)			
**Negative psychological** ** changes associated with the** **start of the hormonal therapy** None Minimal Mild Moderate Severe Very severe	20 (16.2%) 13 (10.6%) 16 (13.0%) 30 (24.4%) 23 (18.7%) 21 (17.1%)			
**Analgesic drugs assumption to** **control the pain** None Rarely Sometimes Often Very often	21 (12.5%) 21 (12.5%) 44 (26.2%) 41 (24.4%) 41 (24.4%)			

^a^ = *t*-test comparisons. ^b^ = χ^2^ test.

**Table 2:** Comparison between women with endometriosis and women without endometriosis on sleep health and psychological wellbeing (= 338).

	Endometriosis (*n* = 169)	Controls (*n* = 169)	*p* Value
**Insomnia (ISI)**			
Mean ± SD	12.96 ± 6.26	9.47 ± 5.99	**<0.001 ^a^**
			
**Insomnia severity (*n*, %)**			**<0.001 ^b^**
Not clinically significant	37 (21.9%)	73 (43.2%)	
Subthreshold	68 (40.2%)	65 (38.5%)	
Moderate	52 (30.8%)	26 (15.4%)	
Severe	12 (7.1%)	5 (3.0%)	
**Sleep Health (tot)**			
Mean ± SD	27.66 ± 7.42	31.96 ± 7.28	**<0.001 ^a^**
**SH_Satisfaction**			
Mean ± SD	6.26 ± 3.09	8.31 ± 2.81	**<0.001 ^a^**
**SH_Vigilance**			
Mean ± SD	6.82 ± 2.71	8.22 ± 2.20	**<0.001 ^a^**
**SH_Timing**			
Mean ± SD	8.54 ± 2.87	8.09 ± 2.80	0.146 ^a^
**SH_Efficiency**			
Mean ± SD	3.38 ± 2.27	4.51 ± 2.46	**<0.001 ^a^**
**SH_Duration**			
Mean ± SD	2.65 ± 1.22	2.83 ± 1.16	0.171 ^a^
**Sleep duration during weekdays 1 (*n*, %)**			0.122 ^b^
Short	103 (60.9%)	95 (56.2%)	
Recommended	63 (37.3%)	74 (43.8%)	
Long	3 (1.8%)	0 (0.0%)	
**Sleep duration during weekends 1 (*n*, %)**			0.784 ^b^
Short	58 (34.3%)	52 (30.8%)	
Recommended	97 (57.4%)	102 (60.4%)	
Long	14 (8.3%)	15 (8.9%)	
**Anxiety (HADS)**			
Mean ± SD	10.53 ± 4.12	7.83 ± 3.91	**<0.001 ^a^**
			
**Anxiety severity (*n*, %)**			**<0.001 ^b^**
Not clinically significant	38 (22.5%)	88 (52.1%)	
Mild	51 (30.2%)	33 (19.5%)	
Moderate	50 (29.6%)	38 (22.5%)	
Severe	30 (17.8%)	10 (5.9%)	
**Depression (HADS)**			
Mean ± SD	8.75 ± 4.08	6.28 ± 3.96	**<0.001 ^a^**
			
**Depression severity (*n*, %)**			**<0.001 ^b^**
Not clinically significant	63 (37.3%)	107 (63.3%)	
Mild	49 (29.0%)	38 (22.5%)	
Moderate	44 (26.0%)	20 (11.8%)	
Severe	13 (7.7%)	4 (2.4%)	
**Emotion Regulation (DERS)**			
Mean ± SD	45.02 ± 14.76	38.20 ± 12.28	**<0.001 ^a^**
**Chronotype (rMEQ)**			
Mean ± SD	14.85 ± 4.07	15.50 ± 3.90	0.131 ^a^
			
**Prevalence (*n*, %)**			0.403 ^b^
Evening type	27 (16.0%)	21 (12.4%)	
Intermediate type	107 (63.3%)	104 (61.5%)	
Morning type	35 (20.7%)	44 (26.0%)	

^1^ = The coding follows “National Sleep Foundation” recommendations (short sleep < 7 h, recommended sleep 7–9 h, long sleep > 9 h). ^a^ = *t*-test comparisons. ^b^ = χ^2^ test. Abbreviations: DERS = Difficulties in Emotion Regulation Scale; HADS = Hospital Anxiety and Depression Scale; ISI = Insomnia Severity Index; rMEQ = Morningness Eveningness Questionnaire reduced; SH = Sleep Health. Significant *p* values are reported in bold.

**Table 3:** Comparisons between women with endometriosis with clinical and nonclinical insomnia symptoms on sleep health and psychological wellbeing.

	ISI > 14 (*n* = 64)	ISI ≤ 14 (*n* = 105)	*p* Value
**Age**			
Mean ± SD	34.41 ± 7.66	34.97 ± 6.65	0.614 ^a^
**Dysmenorrhea**			
Mean ± SD	7.62 ± 2.76	7.00 ± 2.67	0.154 ^a^
Missing	1	2	
**Pelvic pain**			
Mean ± SD	6.03 ± 3.14	4.94 ± 2.82	**0.021 ^a^**
Missing	0	2	
**Dyspareunia**			
Mean ± SD	6.81 ± 2.49	5.24 ± 2.75	**<0.001 ^a^**
Missing	0	1	
**Average pain ^1^**			
Mean ± SD	6.82 ± 2.01	5.74 ± 2.16	**0.002 ^a^**
Missing	1	4	
**Negative psychological effects associated with hormonal therapies ^2^**			
Mean ± SD	2.70 ± 1.91	2.68 ± 1.52	0.954 ^a^
Missing	17	29	
**Frequency of analgesics use ^3^**			
Mean ± SD	2.44 ± 1.33	2.31 ± 1.31	0.536 ^a^
Missing	0	1	
**Sleep Health (tot)**			
Mean ± SD	22.89 ± 5.96	30.56 ± 6.71	**<0.001 ^a^**
**SH_Satisfaction**			
Mean ± SD	4.72 ± 2.66	7.20 ± 2.96	**<0.001 ^a^**
**SH_Vigilance**			
Mean ± SD	5.66 ± 2.54	7.52 ± 2.58	**<0.001 ^a^**
**SH_Timing**			
Mean ± SD	8.28 ± 3.08	8.70 ± 2.73	0.354 ^a^
**SH_Efficiency**			
Mean ± SD	1.91 ± 1.52	4.29 ± 2.19	**<0.001 ^a^**
**SH_Duration**			
Mean ± SD	2.33 ± 1.27	2.85 ± 1.15	**0.007 ^a^**
**Anxiety (HADS)**			
Mean ± SD	12.53 ± 3.81	9.31 ± 3.82	**<0.001 ^a^**
**Depression (HADS)**			
Mean ± SD	10.03 ± 4.19	7.97 ± 3.83	**0.001 ^a^**
**Emotion regulation (DERS)**			
Mean ± SD	50.53 ± 14.96	41.67 ± 13.65	**<0.001 ^a^**
**Chronotype (rMEQ)**			
Mean ± SD	14.02 ± 4.44	15.35 ± 3.75	**0.038 ^a^**

^a^ = *t*-test comparisons. ^1^ = the average score across the three endometriosis-related pain items. ^2^ = describes the negative psychological effects associated with the start of hormonal therapy (from 0 = “none” to 5 = “very severe”). ^3^ = describes the frequency of analgesics used to control endometriosis-related pain (from 1 = “never” to 4 = “very often”). Abbreviations: DERS = Difficulties in Emotion Regulation Scale; HADS = Hospital Anxiety and Depression Scale; ISI = Insomnia Severity Index; rMEQ = Morningness Eveningness Questionnaire reduced; SH = Sleep Health. Significant *p* values are reported in bold.

**Table 4:** Pearson correlations between sleep and psychological wellbeing variables.

Variable	*M*	*SD*	1	2	3	4	5	6	7	8	9	10	11
**1. Age**	34.76	7.02											
**2. rMEQ**	15.17	3.99	0.18 **										
**3. ISI_tot**	11.22	6.36	0.04	−0.19 ***									
**4. HADS_Anxiety**	9.18	4.23	−0.04	−0.11 *	0.51 ***								
**5. HADS_Depression**	7.51	4.20	0.10	−0.13 *	0.45 ***	0.70 ***							
**6. DERS_tot**	41.61	13.98	−0.18 ***	−0.19 ***	0.47 ***	0.61 ***	0.56 ***						
**7. SH_Satisfaction**	7.28	3.12	0.12 *	0.46 ***	−0.59 ***	−0.46 ***	−0.48 ***	−0.53 ***					
**8. SH_Vigilance**	7.52	2.56	0.12 *	0.22 ***	−0.43 ***	−0.48 ***	−0.43 ***	−0.50 ***	0.54 ***				
**9. SH_Timing**	8.32	2.84	0.17 **	0.21 ***	−0.03	−0.00	0.03	−0.05	0.19 ***	0.09			
**10. SH_Efficiency**	3.95	2.43	−0.12 *	0.08	−0.70 ***	−0.45 ***	−0.41 ***	−0.35 ***	0.43 ***	0.25 ***	−0.07		
**11. SH_Duration**	2.74	1.19	−0.10	0.14 *	−0.36 ***	−0.21 ***	−0.22 ***	−0.10	0.18 ***	0.13 *	0.08	0.36 ***	
**12. Sleep Health_tot**	29.81	7.65	0.10	0.39 ***	−0.67 ***	−0.52 ***	−0.49 ***	−0.53 ***	0.82 ***	0.69 ***	0.47 ***	0.61 ***	0.42 ***

* *p* < 0.05. ** *p* < 0.01. *** *p* < 0.001. Abbreviations: DERS = Difficulties in Emotion Regulation Scale; HADS = Hospital Anxiety and Depression Scale; ISI = Insomnia Severity Index; rMEQ = Morningness–Eveningness Questionnaire reduced; SH = Sleep Health.

**Table 5:** Multivariable linear regression models (*n* = 338).

Predictors	Insomnia		Sleep Health		Anxiety Model 1		Anxiety Model 2		Depression Model 1		Depression Model 2	
	β	*p* value	β	*p* value	β	*p* value	β	*p* value	β	*p* value	β	*p* value
Age	0.09	**0.032**	0.02	0.732	−0.03	0.160	−0.02	0.387	0.10	**<0.001**	0.10	**<0.001**
Group (E)	1.25	**0.039**	−1.34	**0.044**	0.64	**0.042**	0.69	**0.032**	0.49	0.142	0.43	0.195
Insomnia	-	-	-	-	0.12	**<0.001**	-	-	0.03	0.334	-	-
Sleep Health	-	-	-	-	-	-	−0.09	**0.001**	-	-	−0.07	**0.014**
Anxiety	0.44	**<0.001**	−0.39	**0.001**	-	-	-	-	0.51	**<0.001**	0.49	**<0.001**
Depression	0.10	0.334	−0.27	**0.014**	0.47	**<0.001**	0.46	**<0.001**	-	-	-	-
Emotion regulation	0.10	**<0.001**	−0.13	**<0.001**	0.08	**<0.001**	0.08	**<0.001**	0.07	**<0.001**	0.06	**<0.001**
Chronotype	−0.19	**0.011**	0.55	**<0.001**	0.05	0.192	0.08	0.059	−0.05	0.245	−0.01	0.727
F	27.85		45.27		79.25		76.79		66.87		68.77	
*p* value	**<0.001**		**<0.001**		**<0.001**		**<0.001**		**<0.001**		**<0.001**	
R^2^	0.34		0.45		0.59		0.58		0.55		0.56	

Note: for anxiety and depression, model 1 includes insomnia (ISI) as a predictor and model 2 includes sleep health (set of questions on sleep health) as a predictor. Significant *p* values are reported in bold. Abbreviations: E = endometriosis group.

**Table 6:** Multiple regression models on psychological outcomes performed on the endometriosis group.

Predictors	Insomnia		Sleep Health		Anxiety Model 1		Anxiety Model 2		Depression Model 1		Depression Model 2	
	β	*p* value	β	*p* value	β	*p* value	β	*p* value	β	*p* value	β	*p* value
**Insomnia**	-	-	-	-	0.11	**0.008**	-	-	0.01	0.915	-	-
**Sleep Health**	-	-	-	-	-	-	−0.08	0.051	-	-	−0.07	0.173
**Anxiety**	0.41	**0.006**	−0.45	**0.007**	-	-	-	-	0.41	**<0.001**	0.37	**<0.001**
**Depression**	0.00	0.982	−0.17	0.288	0.35	**<0.001**	0.34	**<0.001**	**-**	-	-	-
**Emotion regulation**	0.06	0.127	−0.12	**0.006**	0.09	**<0.001**	0.09	**<0.001**	0.08	**0.001**	0.07	**0.006**
**Pain**	0.33	0.132	−0.24	0.334	0.26	**0.020**	0.26	**0.026**	0.11	0.442	0.09	0.533
**Disease stage (II)**	-	-	−1.97	0.229	**-**	**-**	-	-	0.41	0.666	0.30	0.747
**Disease stage (III)**	-	-	−1.24	0.399	-	-	-	-	0.07	0.939	−0.03	0.975
**Disease stage (IV)**	-	-	−3.41	**0.020**	-	-	-	-	1.51	0.067	1.27	0.130
**Chronotype**	−0.21	0.060	0.58	**<0.001**	-	-	-	-	-	-	-	-
**F**	8.98		15.04		43.26		41.55		14.71		15.21	
***p* value**	**<0.001**		**<0.001**		**<0.001**		**<0.001**		**<0.001**		**<0.001**	
**R^2^**	0.22		0.51		0.52		0.51		0.46		0.47	

Note: for anxiety and depression, model 1 includes insomnia (ISI) as a predictor and model 2 includes sleep health (set of questions on sleep health) as a predictor. Models for sleep health and depression as outcomes were run on 127 women, while models for insomnia and anxiety were run on 164 women because of missing data. Significant *p* values are reported in bold.

**Table 7:** Multiple regression models on disease-related pain performed on the endometriosis group.

Predictors	Pain * Model 1		Pain * Model 2	
	β	*p* value	β	*p* value
**Age**	−0.05	0.078	−0.05	0.105
**Insomnia**	0.02	0.587	**-**	**-**
**Sleep Health**	**-**	-	−0.03	0.359
**Anxiety**	0.15	**0.015**	0.14	**0.020**
**Depression**	0.07	0.245	0.06	0.312
**Emotion regulation**	−0.00	0.874	−0.00	0.754
**Disease stage (II)**	1.45	**0.015**	1.41	**0.017**
**Disease stage (III)**	1.17	**0.032**	1.12	**0.039**
**Disease stage (IV)**	1.41	**0.011**	1.32	**0.020**
**Symptoms 4–10 years**	0.06	0.920	0.06	0.926
**Symptoms more than 10 years**	0.68	0.235	0.66	0.247
**F**	4.28		4.36	
***p* value**	**<0.001**		**<0.001**	
**R^2^**	0.27		0.27	

* The average score across the three endometriosis-related pain items (dysmenorrhea, pelvic pain, and dyspareunia). Note: model 1 includes insomnia (ISI) as a predictor, and model 2 includes sleep health (set of questions on sleep health) as a predictor. Models were run on 127 women because of missing data. Significant *p* values are reported in bold.

## Data Availability

The data presented in this study are available on request from the corresponding author due to privacy or ethical restrictions.

## Supplementary Materials

The following supporting information can be downloaded at: https://www.mdpi.com/article/10.3390/jcm14062103/s1, Table S1: Demographic and clinical characteristics of the sample of university students (= 1078); Table S2: Univariate regression models in endometriosis group.
