# Supplementary material for: Sleep Health and Psychological Wellbeing in Adult Women: A Specific Focus on Endometriosis—A Survey Study
Source: J Clin Med. 2025 Mar 19;14(6):2103. doi: 10.3390/jcm14062103 (PMC11942798; doi:10.3390/jcm14062103)
Supplement: Supplementary file 1 [file jcm-14-02103-s001.zip › jcm-3413982-supplementary.pdf]

Table S1. Demographic and clinical characteristics of the total sample of students (= 1078)

	Student sample (n = 1078)
**Age**	
Mean ± SD	30.72 ± 7.57
Age range	18 – 45
**Employment**	
Employed	702 (65.1%)
Not employed	75 (7.0%)
Student	249 (23.1%)
Student-worker	44 (4.1%)
Other	8 (0.7%)
**Relationship status**	
Married or cohabiting	415 (38.5%)
In a relationship	361 (33.5%)
Divorces or separated	26 (2.4%)
Single	275 (25.5%)
Widow	1 (0.1%)
**Education**	
Middle school diploma	1 (0.1%)
High school	538 (49.9%)
Bachelor's degree	317 (29.4%)
Master's degree/single-cycle degree	139 (12.9%)
Post-graduate master's degree	77 (7.1%)
PhD	5 (0.5%)
Other	1 (0.1%)
**Insomnia (ISI)**	
Mean ± SD	9.67 ± 5.94
**Sleep Health (tot)**	
Mean ± SD	31.23 ± 7.31
**SH_Satisfaction**	
Mean ± SD	7.73 ± 2.99
**SH_Vigilance**	
Mean ± SD	8.12 ± 2.30

**SH_Timing**<br>Mean ± SD	7.95 ± 2.86
**SH_Efficiency**<br>Mean ± SD	4.59 ± 2.35
**SH_Duration**<br>Mean ± SD	2.84 ± 1.10
**Anxiety (HADS)**<br>Mean ± SD	8.56 ± 4.27
**Depression (HADS)**<br>Mean ± SD	6.49 ± 3.91
**Emotion Regulation (DERS)**<br>Mean ± SD	40.71 ± 13.57

Abbreviations: DERS = Difficulties in Emotion Regulation Scale; HADS = Hospital Anxiety and Depression Scale; ISI = Insomnia Severity Index; SH = Sleep Health.

Table S2. Univariate regression models in endometriosis group

a) Univariate regression model for insomnia

**Insomnia (ISI)**					
Predictors	*Estimates*	*std. Error*	*Statistic*	*p*	Observations
(Intercept)	14.07	2.44	5.77	**<0.001**	169
Age	-0.03	0.07	-0.46	0.643	
(Intercept)	8.45	1.42	5.96	**<0.001**	164
Average pain	0.75	0.22	3.47	**0.001**	
(Intercept)	12.81	1.37	9.34	**<0.001**	169
Duration of symptoms [4-10 years]	-0.38	1.65	-0.23	0.816	
Duration of symptoms [More than 10 year]	0.44	1.51	0.29	0.772	
(Intercept)	12.04	1.30	9.30	**<0.001**	131
Disease stage [II]	0.66	1.81	0.37	0.714	
Disease stage [III]	0.53	1.67	0.32	0.752	
Disease stage [IV]	2.02	1.57	1.29	0.201	
(Intercept)	6.14	1.20	5.11	**<0.001**	169
HADS A Tot	0.65	0.11	6.09	**<0.001**	
(Intercept)	8.86	1.09	8.13	**<0.001**	169
HADS D Tot	0.47	0.11	4.15	**<0.001**	
(Intercept)	5.78	1.44	4.01	**<0.001**	169
DERS Tot	0.16	0.03	5.26	**<0.001**	
(Intercept)	17.16	1.80	9.53	**<0.001**	169
MEQr Tot	-0.28	0.12	-2.42	**0.017**	

b) Univariate regression model for Sleep Health

**Sleep Health**					
Predictors	*Estimates*	*std. Error*	*Statistic*	*p*	Observations
(Intercept)	24.09	2.88	8.36	**<0.001**	169
Age	0.10	0.08	1.26	0.209	
(Intercept)	35.08	1.62	21.65	**<0.001**	164
Average pain	-1.24	0.25	-4.99	**<0.001**	
(Intercept)	28.81	1.63	17.71	**<0.001**	169
Duration of symptoms [4-10 years]	-1.26	1.96	-0.64	0.522	
Duration of symptoms [More than 10 year]	-1.34	1.79	-0.75	0.453	
(Intercept)	30.78	1.52	20.28	**<0.001**	131
Disease stage [II]	-2.03	2.12	-0.96	0.340	
Disease stage [III]	-2.21	1.95	-1.13	0.260	
Disease stage [IV]	-5.19	1.84	-2.82	**0.006**	
(Intercept)	37.26	1.36	27.39	**<0.001**	169
HADS A Tot	-0.91	0.12	-7.58	**<0.001**	
(Intercept)	34.82	1.21	28.69	**<0.001**	169
HADS D Tot	-0.82	0.13	-6.51	**<0.001**	
(Intercept)	39.40	1.58	25.00	**<0.001**	169
DERS Tot	-0.26	0.03	-7.84	**<0.001**	
(Intercept)	17.02	2.00	8.52	**<0.001**	169
MEQr Tot	0.72	0.13	5.51	**<0.001**	

c) Univariate regression model for anxiety symptoms

Anxiety (HADS)					
Predictors	*Estimates*	*std. Error*	*Statistic*	*p*	Observations
(Intercept)	12.82	1.60	8.03	**<0.001**	169
Age	-0.07	0.05	-1.46	0.146	
(Intercept)	6.33	0.91	6.96	**<0.001**	164
Average pain	0.70	0.14	5.00	**<0.001**	
(Intercept)	9.52	0.90	10.58	**<0.001**	169
Duration of symptoms [4-10 years]	1.11	1.08	1.03	0.305	
Duration of symptoms [More than 10 year]	1.17	0.99	1.18	0.239	
(Intercept)	9.87	0.86	11.52	**<0.001**	131
Disease stage [II]	-0.08	1.20	-0.06	0.948	
Disease stage [III]	0.62	1.10	0.56	0.578	
Disease stage [IV]	1.23	1.04	1.19	0.238	
(Intercept)	6.90	0.66	10.40	**<0.001**	169
ISI Tot	0.28	0.05	6.09	**<0.001**	
(Intercept)	18.29	1.06	17.26	**<0.001**	169
SH Tot	-0.28	0.04	-7.58	**<0.001**	
(Intercept)	5.18	0.60	8.64	**<0.001**	169
HADS D Tot	0.61	0.06	9.83	**<0.001**	
(Intercept)	3.02	0.82	3.69	**<0.001**	169
DERS Tot	0.17	0.02	9.64	**<0.001**	
(Intercept)	11.88	1.20	9.89	**<0.001**	169
MEQr Tot	-0.09	0.08	-1.16	0.247	

d) Univariate regression model for depressive symptoms

**Depression (HADS)**					
Predictors	*Estimates*	*std. Error*	*Statistic*	*p*	*Observations*
(Intercept)	6.99	1.58	4.41	**<0.001**	169
Age	0.05	0.04	1.13	0.259	
(Intercept)	5.28	0.92	5.72	**<0.001**	164
Average pain	0.57	0.14	4.03	**<0.001**	
(Intercept)	8.14	0.89	9.11	**<0.001**	169
Duration of symptoms [4-10 years]	0.64	1.08	0.60	0.550	
Duration of symptoms [More than 10 year]	0.72	0.98	0.73	0.466	
(Intercept)	7.61	0.83	9.14	**<0.001**	131
Disease stage [II]	0.64	1.16	0.55	0.583	
Disease stage [III]	0.48	1.07	0.45	0.657	
Disease stage [IV]	2.39	1.01	2.37	**0.019**	
(Intercept)	6.17	0.69	8.92	**<0.001**	169
ISI Tot	0.20	0.05	4.15	**<0.001**	
(Intercept)	15.59	1.09	14.34	**<0.001**	169
SH Tot	-0.25	0.04	-6.51	**<0.001**	
(Intercept)	2.43	0.69	3.53	**0.001**	169
HADS A Tot	0.60	0.06	9.83	**<0.001**	
(Intercept)	2.04	0.85	2.39	**0.018**	169
DERS Tot	0.15	0.02	8.28	**<0.001**	
(Intercept)	9.80	1.19	8.23	**<0.001**	169
MEQr Tot	-0.07	0.08	-0.91	0.363	

e) Univariate regression model for pain

**Pain**					
Predictors	*Estimates*	*std. Error*	*Statistic*	*p*	Observations
(Intercept)	7.90	0.84	9.45	**<0.001**	164
Age	-0.05	0.02	-2.13	**0.035**	
(Intercept)	5.10	0.47	10.75	**<0.001**	164
Duration of symptoms [4-10 years]	0.78	0.57	1.38	0.170	
Duration of symptoms [More than 10 year]	1.40	0.52	2.69	**0.008**	
(Intercept)	4.90	0.44	11.26	**<0.001**	127
Disease stage [II]	1.74	0.62	2.83	**0.006**	
Disease stage [III]	1.42	0.56	2.53	**0.013**	
Disease stage [IV]	1.74	0.53	3.26	**0.001**	
(Intercept)	4.96	0.38	12.95	**<0.001**	164
ISI Tot	0.09	0.03	3.47	**0.001**	
(Intercept)	9.11	0.61	14.89	**<0.001**	164
SH Tot	-0.11	0.02	-4.99	**<0.001**	
(Intercept)	4.12	0.44	9.43	**<0.001**	164
HADS A Tot	0.19	0.04	5.00	**<0.001**	
(Intercept)	4.75	0.38	12.38	**<0.001**	164
HADS D Tot	0.16	0.04	4.03	**<0.001**	
(Intercept)	4.52	0.53	8.53	**<0.001**	164
DERS Tot	0.04	0.01	3.25	**0.001**	
(Intercept)	6.87	0.64	10.77	**<0.001**	164
MEQr Tot	-0.05	0.04	-1.15	0.250	

Abbreviations: DERS = Difficulties in Emotion Regulation Scale; HADS = Hospital Anxiety and Depression Scale; ISI = Insomnia Severity Index; rMEQ = Morningness-Eveningness Questionnaire reduced; SH = Sleep Health.

Significant p values are reported in bold.
